# Removal of the Cecum for Carcinoma—Recovery1Read at the thirty-fourth annual meeting of the Medical Association of Central New York, held at Buffalo, N. Y., August 27-28, 1901.

**Published:** 1901-10

**Authors:** W. L. Conklin

**Affiliations:** Rochester, N. Y.


### Removal of the Cecum for Carcinoma—Recovery.[1]

By W. L. CONKLIN, M. D., Rochester, N. Y.

CARCINOMA of the cecum is of comparatively rare occurrence, and at the same time possessed of peculiar features which render its diagnosis obscure and its treatment difficult. Ziemssen states that of 9,000 fatal cases of carcinoma only about 4 per cent. were intestinal. While the rectum is involved in 80 per cent. of intestinal carcinomata the cecum is affected in only $4\frac{1}{2}$ per cent. Carcinoma of the cecum, then, occurs in about $\frac{18}{100}$ of 1 per cent. of all cases of carcinoma.

In view of the fact that recent investigations regarding the etiology of cancer seem likely to establish its germ origin we

1. Read at the thirty-fourth annual meeting of the Medical Association of Central New York, held at Buffalo, N. Y., August 27-28, 1901.

may infer that the traumatism which so often precedes cancer of the cecum has acted only as a predisposing cause, by weakening the resisting power of the tissues.

Many of the symptoms of this affection might easily be mistaken for those due to chronic appendicitis, indigestion, or fecal impaction. Pain, loss of flesh and strength, a tumor in the right iliac region and a cachexia, which is perhaps more pronounced in intestinal than in other varieties of cancer, are the most prominent symptoms. The pain is apt to radiate toward the umbilicus or down the thigh, and varies in character and intensity, from a mere feeling of discomfort, during or after meals, to severe and prolonged intestinal colic.

If the growth be of the annular variety, symptoms of intestinal obstruction may develop and suddenly become alarming in character. Vomitus, with a fecal odor or taste points to obstruction, which may be due to intestinal carcinoma. In one reported case such vomiting occurred a year before any tumor could be detected. Concurrent attacks of appendicitis are of not infrequent occurrence, during the course of this disease. Dr. Charles G. Cumston has recently reported a case of Carcinoma of the Cecum, Complicated by Appendicitis. It would seem that in the case, which I am about to report, continued appendicular colic culminated in an attack of appendicitis, with the formation of pus, and that this, in turn, was followed by the development of cancer of the cecum. It is, however, difficult or impossible to determine at what period the element of malignancy became a feature of the case.

The only treatment, other than palliative, consists in the removal of the diseased cecum. Morrison, of Birmingham, Eng., states that the earliest successful case of excision of the cecum which he can find recorded was reported by Billroth in 1883, and that 22 cases were collected by Rolleston and Shield up to the end of 1896. The mortality rate of this series was 50 per cent.

An early diagnosis, before symptoms of serious obstruction appear; the presence of an annular rather than a diffuse growth and the absence of glandular involvement are conditions greatly increasing the probabilities of recovery after operation. Fortunately, glandular involvement is often delayed or absent. Ziemssen says: "Primary cancer of the intestines generally remains as an isolated tumor;" while Mayo states, in a recent paper, that "Glandular infection occurs in less than one-half of the cases dying from this malady."

G. K., grocery clerk, aged 43, was first seen by me in consultation with Dr. Stockschlaeder, June 18, 1900. The following history was obtained: In August, 1899, he had fallen in such a way as to strike the right side of the abdomen against the edge of a box, causing considerable pain. The following January he began to have attacks of abdominal pain, which were attributed to indigestion. Three months later he first consulted Dr. Stockschlaeder. On examination the doctor found a small mass, somewhat tender on pressure, in the region of the appendix. The temperature was normal and pulse nearly so. A marked cachexia was noticed. The patient was informed as to the necessity of remaining quiet and the possible need of operation and sent home. The condition then present improved so that work was soon resumed, and continued until about a week before I was called.

During this illness there had been, up to the day I saw him, but little disturbance of pulse and temperature, but pain had been complained of in the right iliac region and a large tumor could easily be recognised. Operation, though strongly urged, had been refused. Just preceding my visit there had been a prolonged chill, with rapid pulse and rise of temperature. This was followed by profuse perspiration. Extreme pallor was noticed and chronic nephritis suspected, as its cause. It seemed evident that we had to deal with an appendicular abscess with beginning septic infection. An operation was reluctantly consented to, and performed that night.

A large amount of pus was found, walled in by firm adhesions and it did not seem wise to disturb these in an attempt to find and remove the appendix. Threatening symptoms of sepsis disappeared at once and under Dr. Stockschlaeder's care the patient made a rapid recovery, leaving the hospital in about two weeks. The incision was entirely closed, with an apparently firm cicatrix, in about a month.

An attempt was made to resume work, but more or less discomfort was complained of around the scar. Four weeks later a small abscess suddenly developed at one side of the cicatrix, broke and discharged for a few days. A small fistulous opening remained, with slight discharge of thin purulent fluid, having at times a fecal odor.

November 12, the patient was seen by Drs. F. H. Zimmer and Richard Moore in consultation. At this time the extreme pallor, which had been noticed before, still continued, although iron and arsenic had been given freely. There was marked infiltration of the tissues on either side of the fistulous opening. Operation was advised, and on November 14, at the City Hospital, I made an incision near and parallel to the cicatrix and entered what seemed to be the old abscess cavity. In this cavity there was a small amount of pus and considerable necrotic tissue. The cavity was flushed with salt solution and packed, a counter opening having been made in the loin, and a drainage-tube inserted. It was thought advisable to give nature another chance to bring about closure of the fistulous opening in the bowel.

Following this operation there was a discharge of fecal matter, abundant at first but gradually diminishing in amount. The cavity was packed with gauze—a large amount being required for two weeks following the operation. On the sixth day, when patient was using bed-pan, the orderly noticed a strip of gauze in the pan. He cut this off and I was notified by telephone of the somewhat unusual result following the laxative enema. I found one end of the gauze packing protruding from the anus and by gentle traction removed about one and a half feet of gauze. Evidently the packing had found its way through the fistulous opening into the cecum, and I could only account for its appearance in the rectum on the supposition that a fistulous communication had been established between the cecum and rectum.

Although the amount of fecal matter discharged from the fistulous opening diminished for a time, there seemed to be no improvement after about December 15th. There was marked and increasing infiltration of the abdominal wall about the opening. The possibility of this infiltration being due to the development of a malignant growth, was suggested by Dr. F. W. Zimmer. Microscopical examination tended to confirm this opinion and on December 31, with the assistance and counsel of Drs. Zimmer and Moore, and other physicians present, I operated for the removal of this growth and the repair of the bowel.

An elliptical incision was made through the skin, outside the mass of indurated tissue which surrounded the fistulous opening. The infiltration had involved fascia, muscle and peritoneum, making it necessary to sacrifice a large area of abdominal wall. This infiltrated mass was firmly attached to the cecum, which had, through its entire length undergone the same pathological change. Firm and extensive adhesions made its liberation and removal very difficult. An attempt was made to make use of a potato button to facilitate anastomosis of ileum with ascending colon, but without success. Had the button been large at one end and small at the other, corresponding to the difference in caliber of colon and ileum, it might have been of service. Though this difference in caliber increased the difficulty of end-to-end anastomosis, it was finally accomplished by means of the Czerny-Lembert suture. It had been necessary to remove so much peritoneum that it was impossible to bring its edges together for about one-half the length of the incision. Silkworm gut was used for the remaining sutures, but the tension was so great as to make it necessary to remove most of them two or three days later.

On the day following the operation the patient became very much distended. His pulse increased in rapidity until it reached 160, while the temperature was 103°. Failing by other means to relieve the distension, I did an enterostomy in the median line, below the umbilicus, suturing the edges of the intestinal incision to the peritoneum and fascia. This was followed by free escape of gas and fecal matter and immediate relief of distension. The pulse dropped in six hours to 130 and in twenty-four to 112.

Improvement was slow but practically uninterrupted. There was very little, if any, discharge of fecal matter at the point of anastomosis, but the discharge of pus was free and for a time extremely offensive.

In its center the edges of the incision were at least four inches apart, but by a gradual process of granulation and contraction a firm and comparatively narrow cicatrix formed in about eight weeks.

The patient left the hospital nine weeks after the last operation and for six weeks has been following his usual occupation. He is apparently in good health.

Following is the report of Prof. Charles Wright Dodge, of the University of Rochester, to whom the growth was submitted for microscopical examination.

August 6, 1901.

*My Dear Doctor Conklin*:

I find the condition of the cecum sent for examination to be due to the presence of a carcinomatous growth. The mucous membrane of the cecum itself is entirely eroded and the colon is becoming involved. Very truly yours,

CHARLES WRIGHT DODGE.

720 SOUTH AVE.

DISCUSSION.

Dr. F. S. HIGGINS, Cortland.—It seems these cases must be somewhat more common than Dr. Conklin's figures would indicate. I understood him there is only .18 of 1 per cent. reported. I must think some of this is due to difficulty of diagnosis. I had one very hard case, which I was unsuccessful with. It seems to me in diagnosis, the age is important, the temperature is important, being so low in the cancerous and normal in appendicitis. These points, it seems to me, will help us out in the future. In my own case the woman was past fifty. I can't help but think, too, that some of these cases that are simply inflammatory have been called cancerous. I know a doctor who came to our county and operated on a man with all the signs of cancer, and it was so reported by the microscopist, and the patient got well. It seems to me one of two things must be true. If it is cancerous you can't remove it; if it is inflammatory, with or without an operation, he may possibly recover. In this case, of course, the report of the microscopist would show there was cancer. I am sure it must be very difficult, as they infiltrate deep into the muscles, and in the case I had way back into the hip-joint.

